# The Relationship Between Spirituality, Stress, and Depression Among Health Professionals in Greece

**DOI:** 10.3390/healthcare13131484

**Published:** 2025-06-20

**Authors:** Evangelos C. Fradelos, Maria Saridi, Vasiliki Kitsiou, Anastasios Christakis, Pavlos Sarafis, Ioanna V. Papathanasiou, Dimitra Latsou, Theodosios Paralikas, Aikaterini Toska

**Affiliations:** 1 Department of Nursing, University of Thessaly, 41500 Larissa, Greece; msaridi@uth.gr (M.S.); tassos.christakis@gmail.com (A.C.); psarafis@uth.gr (P.S.); iopapathanasiou@uth.gr (I.V.P.); paralikas@uth.gr (T.P.); ktoska@uth.gr (A.T.); 2 Department of Social & Educational Policy, University of Peloponnese, 22131 Corinth, Greece; vicky_berg@yahoo.com (V.K.); demilatsou@yahoo.gr (D.L.); 3 Department of Economics and Business, Neapolis University, Pafos 8042, Cyprus

**Keywords:** spirituality, stress, depression, healthcare professionals, mental health

## Abstract

**Background**: Spirituality has emerged as a potential protective factor that may promote mental well-being and resilience among healthcare workers. **Aim**: This study aims to examine the relationship between spirituality, stress, and depression among healthcare professionals in Greece. **Methods**: This cross-sectional study surveyed 412 employees at the Corinth General Hospital in Greece, including medical, nursing, and administrative personnel. The data were collected using the Functional Assessment of Chronic Illness Therapy—Spiritual Well-Being 12 (FACIT-SP12), the Perceived Stress Questionnaire (PSQ), and the Center for Epidemiologic Studies Depression Scale (CES-D). The statistical analyses included non-parametric tests, correlation coefficients, and multiple regression. **Results**: The mean spirituality score was 34.6 (±6.83), while the stress and depression scores were 74.6 (±14.87) and 14.7 (±10.20), respectively. Spirituality was significantly and negatively correlated with both stress (r = −0.479, *p* < 0.001) and depression (r = −0.452, *p* < 0.001). Gender, years of service, educational level, and marital status were also associated with variations in stress, depression, and spirituality levels. Women and those with lower education reported significantly higher levels of stress and depression. Additionally, some demographic variables such as age and sector of employment did not show significant associations with spirituality or depression. The regression analysis confirmed spirituality as an independent predictor of lower stress levels (B = −1.158, *p* < 0.001). **Conclusions**: Spirituality is a significant predictor in mitigating stress and depression among healthcare workers. Promoting a supportive spiritual climate and incorporating elements of spiritual leadership in healthcare settings may enhance employee well-being and resilience. Future research should expand on these findings across diverse institutional and cultural contexts.

## 1. Introduction

Healthcare professionals encounter various challenges that test their mental endurance. Key issues include staff shortages, long and exhausting shifts, caring daily for patients with severe or chronic illnesses, communicating with patients’ relatives [1], interpersonal conflicts, and organizational barriers [2]. These factors significantly affect the mental health of public hospital employees. Working in a high-intensity environment while dealing with the pain and death of others increases the risk of developing health problems, including psychological issues, which often remain unreported due to fears of stigmatization [3]. This situation creates an impasse for employees, affecting their work behavior, relationships with colleagues, and the care provided to patients.

Achieving inner harmony through spirituality can provide employees with a sense of personal satisfaction and enhance their resilience in adverse circumstances, thereby improving both their mental health and performance [4]. Spirituality, while often associated with religion, is a broader and deeply personal experience that reflects each individual’s search for meaning, purpose, inner balance, and transcendence [5,6]. According to Douglas MacDonald, spirituality is a multidimensional construct that includes elements such as existential well-being, spiritual experiences, cognitive orientation toward the transcendent, and, in some cases, religiosity [7]. It has been shown to support psychological resilience, strengthen coping mechanisms, and foster social connectedness, contributing significantly to both individual and collective well-being [6,7].

In a holistic approach to care, it is crucial to consider not only the physical and social aspects of a person but also their spiritual dimension. Recent developments in health sciences reflect a growing interest in spirituality, highlighting its role in crisis management and overall well-being, thereby making it a critical component of holistic care [8].

Daily, healthcare workers face challenges and difficulties that can elevate stress levels and affect their mental resilience. Such prolonged exposure to stress can lead to health problems like depression, other psychiatric disorders, substance abuse, insomnia, digestive issues, headaches, isolation, suicidal thoughts, and other conditions that significantly lower their quality of life [9]. The intensity of stress among healthcare workers is influenced by external factors and endogenous parameters such as personality, experiences of stressful situations, and coping mechanisms [1].

Prolonged stress can lead to anxiety and depression syndromes [9]. Depression, a common mental disorder, is characterized by sadness, a loss of interest in activities, diminished self-esteem, feelings of guilt, thoughts of death, sleep disturbances, and psychotic disorders [10]. Several factors contribute to depression among healthcare professionals, including stressful work environments and the unique demands of their profession, such as rotating shift schedules, poor sleep quality, and gender-related challenges, particularly for women [11]. Other contributing factors include low education and older age [12].

Additionally, individuals with lower socioeconomic status [13] and those who are divorced or single [14] are more likely to experience depression. The prevalence of depression is notably higher among healthcare professionals, especially those working in hospitals [15].

Compared with most other European countries surveyed, a higher percentage of people in Greece (80%) consider religion at least somewhat important. Despite being a secular state with a constitution that seemingly upholds the separation of church and state, Greece remains deeply influenced by the Christian Orthodox faith. A higher percentage of people in Greece (80%) regard religion as at least somewhat important compared with any other European nation [16].

Spirituality is also linked to ego transcendence and perceptions of society [17]. Studies have shown that spirituality and religiosity significantly reduce levels of depression and stress among health professionals, who often work under challenging conditions that lead to stress-related issues [18,19]. Considering the above, this study aims to assess the impact of spirituality on stress and depression in healthcare workers.

## 2. Materials

Participants: employees from all services (medicine, nursing, administration, etc.) of Corinth General Hospital were included in this study.

The demographic characteristics of the sample are presented in Table 1.

Table 2 outlines the occupational characteristics of the sample.

### 2.1. Procedure

The survey lasted from December 2023 to January 2024. A total of 537 questionnaires were distributed, of which 414 were returned, resulting in a response rate of 77%. Data was collected using convenience sampling, and participants completed a structured questionnaire independently. Two questionnaires were excluded due to missing critical data required for scoring, leaving a final total of 412 questionnaires. The distribution process was conducted in hard copy at the workplace over approximately three months.

Measurements: The Functional Assessment of Chronic Illness Therapy—Spiritual Well-Being 12 (FACIT-SP12) Scale—Non-Illness, created by Cella et al. in 1993 [20] and translated into Greek by Fradelos in 2021 [21], was used to measure spiritual well-being levels. This tool provides a total spirituality score (ranging from 0 to 48) and calculates separate scores for three subscales: meaning, faith, and serenity (each ranging from 0 to 16). According to its creators, higher scores indicate greater spiritual well-being and a better quality of life. The “meaning” subscale assesses the extent to which individuals experience a sense of meaning and purpose in their lives; the “peace” subscale evaluates peace and inner harmony; and the “faith” subscale measures the degree to which a person finds strength or comfort in their religious or spiritual beliefs. The Cronbach’s a of the Greek version of the scale is a = 0.77.

Perceived stress levels were measured using the Perceived Stress Questionnaire (PSQ), developed by Levenstein et al. in 1993 [22] and translated into Greek by Karatza et al. in 2014 [23]. The Cronbach’s a of the Greek version of the scale is a = 0.90.

Depression levels were assessed using the Center for Epidemiologic Studies Depression Scale (CES-D), developed by Radloff in 1977 [24] and translated into Greek by Fountoulakis et al. in 2001 [25]. The Cronbach’s a of the Greek version of the scale is a = 0.95.

Additionally, eight items were included to collect demographic and work-related data from participants.

### 2.2. Statistical Analysis

Total scores for all scales were computed, including subscale scores for spirituality (meaning, peace, and faith). Descriptive statistics, such as mean values and percentage distributions, were applied to present the sample.

Normality was tested using the Kolmogorov–Smirnov and Shapiro–Wilk tests and, since none of the variables followed a normal distribution, non-parametric tests were chosen. The Mann–Whitney test was used to investigate statistical differences between total scores and independent variables with two categories, while the Kruskal–Wallis test was applied for variables with three or more categories. The Pearson correlation coefficient was used to test the correlation between scale scores and other continuous variables. Lastly, a multiple regression analysis modeled the relationship between stress (as the dependent variable) and spirituality, demographic, and occupational characteristics (as independent variables). A statistical significance level of *p* = 0.05 was applied to all tests. The statistical software IBM SPSS Statistics Version 25 was used for data analysis.

### 2.3. Ethics

This study was conducted with approval from the Scientific Council of Corinth General Hospital (approval number 31062/18-12-2023). Informed consent was obtained from each participant, and their right to freely and voluntarily withdraw from the research was upheld. Data confidentiality and participant anonymity were strictly maintained throughout the study, and all responses were coded and stored without any personally identifiable information.

## 3. Results

### Demographic and Occupational Characteristics of the Sample

The study sample consisted of 412 healthcare professionals employed at Corinth General Hospital. The majority were women (76.7%), with a mean age of 46.6 years (SD = 9.62). Most participants were married (59.7%) and had children (67.4%). In terms of education, 64.5% held a university or postgraduate degree. Occupationally, nearly half of the participants were nurses (49.0%), followed by physicians (28.9%) and administrative staff (14.8%). The majority were employed in the pathological (49.8%) and surgical (40.3%) sectors, and most had over 20 years of professional experience (45.3%).

Table 3 presents the descriptive statistics for the main measurement scales used in the study: the FACIT-Sp12 (Spiritual Well-Being), the Perceived Stress Questionnaire (PSQ), and the Center for Epidemiologic Studies Depression Scale (CES-D). The mean score of the spirituality scale in this sample is μ = 34.6 ± 6.83, with subscale scores of μ = 13.8 ± 2.29 for meaning, μ = 9.7 ± 3.12 for peace, and μ = 11.1 ± 3.75 for faith. The average value of the PSQ is μ = 74.6 ± 14.87, while the CES-D score averages μ = 14.7 ± 10.20.

Table 4 shows the mean and standard deviation for each characteristic, along with group differences and correlations between the independent variables (demographic and occupational characteristics) and spirituality scores (total and subscales). Education level emerged as the only significant factor affecting the total spirituality score (*p* = 0.003), with individuals holding a master’s degree scoring the highest and those with the lowest education level scoring the lowest.

Moreover, statistically significant relationships were observed between the meaning subscale and marital status (*p* = 0.001), with divorced individuals scoring the highest, and the number of children (*p* = 0.000), where participants with children scored much higher. Education level was the only determinant for the peace subscale (*p* = 0.004), with individuals holding a master’s degree scoring the highest. For the faith subscale, a significant correlation was found with age (*p* = 0.030), education level (*p* = 0.009), and years of service (*p* = 0.032). Older individuals were revealed to have higher levels of faith, as were individuals with lower education and greater years of service.

In Table 5, the mean and standard deviation for each characteristic can be found, as well as the results of testing group differences and correlations between the independent variables and the stress perception and depression scales. It is important to note that gender (*p* = 0.001) played a significant role in shaping stress levels, with women scoring significantly higher than men.

Another factor contributing to higher stress levels was having children (*p* = 0.028). Stress levels also varied by work sector (*p* = 0.029), with higher levels noted among professionals working in operating rooms. Years of service (*p* = 0.010) also influenced perceived stress levels.

Gender (*p* = 0.016) was found to be a significant determinant of depression scores, with women reporting higher levels of depression. Moreover, educational level (*p* = 0.046) was associated with depression, as lower education levels correlated with higher depression scores.

Table 6 highlights the results of the test examining the relationship between spirituality, stress perception, and depression scores. The findings indicated that spirituality was moderately and negatively correlated with stress perception (*p* = 0.000, r = −0.479) and depression (*p* = 0.000, r = −0.452). Additionally, stress perception and depression demonstrated a strong and positive correlation (*p* = 0.000, r = 0.674).

Table 7 summarizes the results of a multiple regression analysis, with the stress score as the target variable and predictors including spirituality score, demographic characteristics, and occupational factors. The R-squared value was 0.317, with an F-statistic of 15.192 and a *p*-value less than 0.005 (F (9, 294) = 15.192, *p* < 0.005).

In this model, gender is treated as a categorical variable, represented by the value 1 for men and 2 for women. The unstandardized coefficient (B) for gender is 3.897, indicating that women report higher levels of stress than men. Conversely, the coefficient (B) for spirituality score is −1.158, suggesting that an increase in spirituality score is associated with a decrease in stress score.

## 4. Discussion

This study assessed stress and depression levels among healthcare professionals and explored their relationship with spirituality. The study highlighted the significant relationship between spirituality and the mental health of healthcare professionals, confirming that higher levels of spirituality were associated with lower levels of stress (r = –0.479, *p* = 0.000) and depression (r = –0.452, *p* = 0.000). The most important factor influencing overall spirituality was educational level (*p* = 0.003), with those with a master’s degree showing the highest levels. At the same time, marriage (*p* = 0.001) and number of children (*p* = 0.000) were associated with higher levels of meaning in life, while age (*p* = 0.030), years of service (*p* = 0.032), and lower educational level (*p* = 0.009) were associated with faith, indicating that older and more experienced workers are more likely to rely on religious or spiritual values.

In contrast, stress and depression were influenced by factors such as gender, marital status, and work environment. Women (*p* = 0.001) and parents (*p* = 0.028) had higher levels of perceived stress, while working in surgical fields (*p* = 0.029) and more years of service (*p* = 0.010) increased stress. In addition, women (*p* = 0.016) and individuals with lower educational levels (*p* = 0.046) had higher levels of depression. The multiple regression analysis showed that spirituality is a determining factor in reducing perceived stress, as increasing spirituality is associated with decreasing stress (–1.158, *p* = 0.000). At the same time, gender was found to affect stress, with women showing higher levels (+3.897, *p* = 0.035).

When comparing these findings on overall spirituality and its dimensions (meaning, peace, and faith) to the study by Fradelos [21], several similarities emerge. The overall spirituality score was similar, with a mean of 34.46 (±7.29) in that study and 34.6 (±6.83) in our study. In the “Meaning” dimension, a comparable score was observed (14.12 ± 2.17 vs. 13.8 ± 2.29), suggesting that participants in both studies found a similar level of meaning in their lives. In “Peace,” the scores were likewise similar (9.93 ± 3.29 vs. 9.7 ± 3.12), while in “Faith,” that study reported a slightly lower score (10.40 ± 4.13 vs. 11.1 ± 3.75). Despite the differences in context and time, both studies involved Greek populations that share religious and spiritual beliefs.

According to the same research by Fradelos [21] on nursing staff in two public hospitals in Athens, gender and family status were two important factors related to spirituality. Notably, the “Peace” dimension statistically differed between men and women, with men having higher values than women. This finding challenges the prevailing view that women are more religious and spiritual, primarily due to greater participation in religious activities like attending church. Recent studies suggest that spirituality or religiosity is no longer inherently linked to gender, highlighting the influence of cultural contexts and different measurement tools. This indicates the need for further investigation and unified assessment tools to draw more reliable conclusions [21,26,27].

The lower “Meaning” scores among single participants align with the existing literature emphasizing the role of family in spirituality and meaning making. According to Steger and Frazier [28], close relationships, such as those found within a family, contribute significantly to the formation of meaning and are positively associated with psychological well-being. Simultaneously, George et al. [29] suggest that family and religious beliefs provide a stable framework for interpreting life, enhancing coherence and purpose. These observations affirm the importance of social connections and family relationships on intellectual development, thus warranting further investigation.

A negative relationship between education and faith has been corroborated by several studies [30]. For instance, Hungerman [31] demonstrated that higher levels of education are associated with lower levels of religious beliefs later in life. Specifically, each additional year of education reduces the likelihood of religious identification by 4 percentage points. Education fosters critical thinking and skepticism, which can challenge traditional religious explanations [32]. At the same time, greater exposure to different worldviews and cultures can broaden individuals’ thinking, leading to a more secular worldview [33]. Furthermore, education promotes autonomy and individualism, decreasing the need for religious guidance [34] while fostering liberal social values that may conflict with traditional religious understandings [35]. Finally, the scientific perspectives offered by education may replace the religious interpretations of existential issues, leading to lower levels of faith [36]. These factors explain why education affects religious beliefs without completely severing individuals from religion, instead promoting a more nuanced view of life. Further exploration of this relationship is needed.

Furthermore, according to recent studies, people tend to become more religious as they age. They may experience less control over their lives, and turn to religion to cope with illness, fear of death, anxiety, and depression. This can lead to increased life satisfaction [37]. Similarly, Koenig et al. [38] report that older adults often report greater religious involvement, suggesting that religion may act as a psychological tool to cope with the uncertainties that accompany aging. The relationship between age and religiosity is complex, with religiosity providing not only comfort but also a means to search for meaning in later life. As people age, their spiritual engagement can become an important coping mechanism that promotes emotional well-being and life satisfaction. The connection between faith and quality of life was also highlighted in a study conducted by Tolentino et al. [39], which focused on health workers in Brazil. Other related studies have shown that individuals with low levels of faith, religiosity, and spirituality tend to have a lower quality of life and less satisfaction [40].

Interestingly, certain demographic and occupational variables, such as age, hospital sector, and years of service, did not show statistically significant associations with spirituality or depression. This suggests that the protective influence of spirituality may operate independently of career stage or workplace setting. These findings highlight the potential universality of spirituality as a psychological resource across professional contexts [38].

Stress levels were found to positively correlate with gender and parental status, with women and parents reporting higher stress. Studies have reported high levels of stress among women due to juggling multiple roles in their family and professional life; the added responsibilities of having children further exacerbate this [41]. The workers in the surgical sector reported the highest stress levels—a correlation that was consistent with international findings [42]. This higher incidence of stress can be explained by several factors related to their professional environment. Surgical workers often operate under extreme pressure, as they are responsible for situations where mistakes can have serious consequences for patients [43]. The fast pace and intensity, combined with immediate and precise decision making during surgical procedures, significantly contributes to stress [44]. Additionally, long shifts, the physical and psychological strain of the job, and the emotional burden associated with life-and-death situations intensify employee stress [45]. When these factors combine with workers’ need for undivided attention and technical skill, it makes the surgical environment particularly more stressful than other areas of medicine.

According to a survey conducted by Wilson et al. [41], females are twice as likely than males to develop moderate or high-level anxiety and depression symptoms that require treatment. Conversely, a study conducted by Fond et al. [46] on health professionals working in public and private healthcare structures in France showed that work environment and professional factors such as burnout, workplace mobbing, and non-participation in decision making can lead to clinical depression. The hospital environment is also mentioned as an aggravating factor in other studies, such as the one conducted by Letvak et al. [15] on nurses, which showed that those employed in hospitals have higher rates of depressive symptoms compared with national norms.

The finding that women reported significantly higher stress and depression levels than men may reflect the cumulative impact of role strain, as many female healthcare workers simultaneously manage professional responsibilities and caregiving duties at home. This is consistent with gender-based stress models, which indicate that women are more likely to experience emotional burden due to sociocultural expectations and work–family conflicts [47].

The present study also highlighted a negative relationship between educational level and depression in healthcare workers; lower educational levels are associated with higher scores on the depression scale. This finding is consistent with other studies [48]. The negative relationship between education and depression can be explained by the fact that healthcare professionals with higher education possess better coping mechanisms for stress and work-related pressures. Education promotes emotional and mental empowerment, and more educated healthcare professionals are more likely to implement self-care strategies and recognize signs of mental health difficulties such as depression [49]. Conversely, workers with lower levels of education may lack the ability or resources to manage their stress, which may exacerbate depressive symptoms [50]. Education can also provide individuals with greater job satisfaction and a sense of control, which helps to reduce mental stress and enhance well-being. The findings on the depression scale are significant and highlight the need to extend psychological support to employees. They also indicate the need for improving working conditions and the work environment, which can adversely affect both physical and psychological health, increasing the risk of depression.

Finally, this study highlighted the connection between spirituality and stress perception, as well as between spirituality and depression. Higher levels of spirituality were found to be associated with lower perceived levels of stress and depression. The existing literature suggests that regular spiritual experiences help individuals cope better with negative emotions and enhance positive ones [51], which may explain these findings. Furthermore, the findings of the present study are corroborated by Zolfaghary et al. [52] in their research of 143 midwives in Iran. Therefore, levels of spirituality are inversely related to levels of stress and depression. The results of various studies showed that spirituality is an important protective factor against anxiety and depression among health professionals in Greece. However, it is important to note that the influence of spirituality may also be shaped by the nature and quality of religious beliefs. For instance, Uludag and Zhao (2024) highlighted how certain forms of religious delusions may negatively affect mental health in patients with schizophrenia [53]. This underlines the need to distinguish between adaptive spirituality and the pathological expressions of religiosity in future research. Spirituality should not be assumed to be universally beneficial; rather, its psychological effects may vary based on content, intensity, and personal or cultural context.

A healthcare organization’s spiritual climate refers to the set of values, practices, and leadership that promotes employees’ connection to the meaning and purpose of their work. A supportive spiritual environment can reduce stress and depression, enhancing the resilience of healthcare professionals, as confirmed by the results of our study. When leaders promote spiritual values such as ethics, empathy, and solidarity, they enhance the staff’s mental well-being and their commitment to their work [54]. Conversely, the lack of such a climate can limit the benefits of individual spirituality, increasing burnout. Therefore, the development of a spiritual work environment is a critical factor in improving the mental health of healthcare professionals and the quality of care provided.

Spiritual leadership in healthcare is a modern approach to management that focuses on creating a supportive work environment where healthcare professionals feel their work has meaning and purpose. Spiritually oriented leaders foster a sense of community, inspire trust, and enhance employee resilience, thereby helping to reduce stress and depression [55]. The results of this study confirm the positive association of spirituality with mental health, as higher levels of spirituality were associated with lower levels of stress and depression. This highlights the importance of spiritual leadership, which can play a critical role in supporting health workers. Leaders who incorporate spirituality into management not only enhance job satisfaction but also promote overall employee well-being, making them more resilient to the challenges of the profession [18]. Therefore, integrating spiritual leadership into management practices can be an effective strategy for improving the mental health of healthcare professionals, reducing stress and depression, and enhancing a sense of purpose and meaning in their work.

While leadership plays an important role in fostering a values-based organizational culture, the operationalization of spirituality to support healthcare professionals’ well-being requires specific, inclusive strategies beyond managerial rhetoric. One practical approach involves offering access to optional mindfulness-based stress reduction (MBSR) programs, reflective practice groups, and narrative medicine sessions, which encourage staff to process emotional experiences, reconnect with the meaning in their work, and cultivate inner calm. These interventions have been associated with reductions in burnout, enhanced resilience, and greater job satisfaction [56,57]. Creating dedicated quiet or reflection spaces within the hospital can also promote emotional balance by providing a physical environment where professionals can pause, meditate, or reflect—particularly important in high-intensity settings. Furthermore, access to spiritual care professionals, not limited to religious chaplains but inclusive of non-denominational support, can offer a valuable outlet for staff coping with moral distress, grief, or compassion fatigue [56,57].

Future studies should adopt longitudinal or experimental designs to assess causality and intervention effectiveness. For instance, implementing structured spiritual support programs (e.g., reflective practice groups, chaplain-led sessions, or mindfulness workshops) and evaluating their impact on stress and well-being could provide actionable insights for healthcare organizations. Qualitative research could also help explore the subjective meaning of spirituality among diverse staff populations.

### Limitations

This study has several limitations. First, its cross-sectional design precludes causal inferences regarding the relationships between spirituality, stress, and depression. Second, the use of convenience sampling from a single hospital limits the generalizability of the findings to other healthcare settings or regions. Moreover, the reliance on self-report questionnaires may have introduced socially desirable answers, considering the nature of the study.

## 5. Conclusions

Healthcare professionals experience high levels of anxiety, stress, and depression due to emotionally demanding work environments. This cross-sectional study among 412 healthcare workers in Greece demonstrated that higher levels of spirituality are associated with lower levels of perceived stress and depression. Specifically, spirituality was found to be a predictor of reduced stress, even when controlling demographic and occupational variables. Women, individuals with lower education levels, and those working in high-intensity sectors (e.g., surgical wards) reported notably higher levels of stress and depression. Conversely, spirituality acted as a protective factor, offering healthcare workers a sense of calm, hope, and resilience. These findings highlight the importance of integrating spiritual resources into healthcare systems to support the mental health of staff.

Future research should focus on longitudinal and interventional studies to examine causal pathways and assess the effectiveness of spirituality-oriented interventions in reducing psychological distress. Such efforts could contribute to a more holistic approach to occupational mental health and promote sustainable well-being among healthcare workers. By addressing the psychological needs of staff through evidence-based, spirituality-informed interventions, healthcare organizations can not only reduce stress and depression but also enhance the overall resilience, satisfaction, and performance of their workforce.

## Figures and Tables

**Table 1:** Demographic characteristics.

Characteristics	N 412 (% on Valid)
Gender	
Male	96 (23.3%)
Female	316 (76.7%)
Age	46.6 (9.62) *
Marital Status	
Married	246 (59.7%)
Cohabitation	18 (4.4%)
Divorced	40 (9.7%)
Single	98 (23.8%)
Other	10 (2.4%)
Children	
No children	134 (32.5%)
1–2 children	207 (50.2%)
More than 2 children	71 (17.2%)
Educational Level	
Primary education	11 (2.7%)
Secondary education	135 (32.8%)
Higher education	155 (37.6%)
Master’s degree	111 (26.9%)

* Mean (standard deviation).

**Table 2:** Occupational characteristics.

Characteristics	N 412 (% on Valid)
Service at the Hospital	
Medicine	119 (28.9%)
Nursing	202 (49.0%)
Administrative	61 (14.8%)
Technical	17 (4.1%)
Emergency center	13 (3.2%)
Sector	
Pathological	156 (49.8%)
Surgical	126 (40.3%)
9.66 ± 2.9711.44 ± 3.60 Medical service (except doctors)	31 (9.9%)
Years of Service	
Less than 5 years	94 (22.9%)
5 to less than 10 years	45 (10.9%)
10 to less than 15 years	39 (9.5%)
15 to less than 20 years	47 (11.4%)
20 or more years	186 (45.3%)

**Table 3:** Descriptive statistics of scales used in the study.

Scale	Mean (SD)
Spirituality Scale (FACIT-SP12)	34.6 (6.83)
Meaning	13.8 (2.29)
Peace	9.7 (3.12)
Faith	11.1 (3.75)
Perception of Stress Scale (PSQ)	74.6 (14.87)
Depression Scale (CES-D)	14.7 (10.20)

**Table 4:** Bivariate analysis: group differences and correlations of spirituality.

Independent Variables	Spirituality		Meaning		Peace		Faith	
	Mean ± SD	Sig.	Mean ± SD	Sig.	Mean ± SD	Sig.	Mean ± SD	Sig.
Gender		0.701		0.311		0.139		0.055
Male	34.75 ± 6.09		14.02 ± 2.09		10.21 ± 2.92		10.52 ± 3.73	
Female	34.51 ± 7.04		13.69 ± 2.35		9.58 ± 3.17		11.24 ± 3.74	
Age	0.062 *	0.216	−0.033 *	0.508	0.030 *	0.553	0.109 *	0.030
Marital status		0.107		0.001		0.591		0.271
Married	34.73 ± 6.55		14.00 ± 2.14		9.65 ± 3.01		11.08 ± 3.80	
Cohabitation	33.11 ± 8.35		13.06 ± 2.84		9.61 ± 4.00		10.44 ± 3.84	
Divorced	36.78 ± 6.44		14.43 ± 2.07		10.42 ± 3.14		11.93 ± 3.61	
Single	33.83 ± 6.90		13.13 ± 2.43		9.72 ± 3.11		10.97 ± 3.63	
Other	31.50 ± 9.58		12.90 ± 2.85		9.00 ± 4.45		9.60 ± 3.92	
Children		0.069		0.000		0.710		0.112
No children	33.43 ± 7.28		13.14 ± 2.45		9.58 ± 3.34		10.70 ± 3.68	
1–2 children	34.95 ± 6.28		14.08 ± 2.11		9.76 ± 2.89		11.11 ± 3.65	
More than 2 children	35.59 ± 7.25		14.04 ± 2.29		9.90 ± 3.38		11.65 ± 4.09	
Educational Level		0.003		0.057		0.004		0.009
Primary education	33.09 ± 6.89		13.00 ± 2.00		9.00 ± 2.83		11.09 ± 4.16	
Secondary education	35.36 ± 7.32		13.70 ± 2.52		9.82 ± 3.30		11.84 ± 3.70	
Higher education	33.19 ± 6.56		13.57 ± 2.25		9.10 ± 3.10		10.51 ± 3.66	
Master’s degree	35.68 ± 6.26		14.20 ± 2.04		10.56 ± 2.79		10.92 ± 3.79	
Service at the Hospital		0.321		0.337		0.906		0.053
Medicine	33.87 ± 6.87		13.79 ± 2.27		9.76 ± 3.32		10.32 ± 3.82	
Nursing	35.22 ± 6.79		13.76 ± 2.29		9.83 ± 3.10		11.63 ± 3.43	
Administrative	34.33 ± 6.58		13.93 ± 2.37		9.44 ± 2.99		10.95 ± 4.02	
Technical	32.00 ± 8.25		12.82 ± 2.46		9.12 ± 3.18		10.06 ± 4.26	
Emergency center	35.31 ± 5.60		14.15 ± 2.03		10.00 ± 2.42		11.15 ± 4.78	
Sector		0.309		0.538		0.547		0.086
Pathological	34.89 ± 6.42		13.95 ± 2.11		9.85 ± 3.19		11.09 ± 3.48	
Surgical	34.67 ± 7.07		13.57 ± 2.42		9.66 ± 2.97		11.44 ± 3.60	
Medical service (except doctors)	33.03 ± 6.80		13.84 ± 2.05		9.48 ± 3.58		9.71 ± 4.11	
Years of Service		0.070		0.882		0.199		0.032
Less than 5 years	33.31 ± 7.32		13.66 ± 2.29		9.40 ± 3.51		10.24 ± 4.00	
5 to less than 10 years	34.60 ± 7.41		13.82 ± 2.24		10.09 ± 2.88		10.69 ± 3.81	
10 to less than 15 years	33.26 ± 7.31		13.79 ± 2.41		9.10 ± 3.08		10.36 ± 4.22	
15 to less than 20 years	36.64 ± 6.24		14.06 ± 2.21		10.49 ± 2.91		12.09 ± 3.49	
20 or more years	34.98 ± 6.34		13.73 ± 2.33		9.75 ± 3.03		11.50 ± 3.45	

* Pearson’s Correlation Coefficient.

**Table 5:** Group differences and correlations of stress and depression.

Independent Variables	PSQ		CES_D	
	Mean ± SD	Sig.	Mean ± SD	Sig.
Gender		0.001		0.016
Male	70.39 ± 14.52		12.51 ± 9.10	
Female	75.87 ± 14.76		15.41 ± 10.44	
Age	0.087 *	0.084	−0.020 *	0.690
Marital Status		0.075		0.952
Married	76.05 ± 14.01		14.59 ± 10.12	
Cohabitation	76.56 ± 10.78		13.44 ± 9.39	
Divorced	73.00 ± 12.86		14.64 ± 8.93	
Single	71.13 ± 17.08		15.42 ± 11.06	
Other	75.30 ± 21.51		14.10 ± 11.26	
Children		0.028		0.773
No children	71.83 ± 16.36		15.16 ± 10.56	
1–2 children	76.18 ± 13.87		14.43 ± 9.50	
More than 2 children	75.17 ± 14.18		14.80 ± 11.57	
Educational Level		0.176		0.046
Primary education	78.64 ± 13.79		18.45 ± 9.29	
Secondary education	73.92 ± 15.384		15.82 ± 11.87	
Higher education	76.27 ± 14.64		15.14 ± 9.64	
Master’s degree	72.66 ± 13.90		12.46 ± 8.59	
Service at the Hospital		0.089		0.979
Medicine	74.52 ± 14.70		14.16 ± 9.52	
Nursing	73.47 ± 15.18		15.20 ± 11.21	
Administrative	79.36 ± 13.85		14.52 ± 8.88	
Technical	73.59 ± 13.93		14.29 ± 9.25	
Emergency center	71.54 ± 14.90		14.15 ± 7.29	
Sector		0.029		0.007
Pathological	71.87 ± 14.44		13.08 ± 9.12	
Surgical	34.67 ± 7.07		13.57 ± 2.42	
Medical service (except doctors)	33.03 ± 6.8013.84 ± 2.059.48 ± 3.589.71 ± 4.11 * Pearson correlation coefficient 76.40 ± 15.6217.49 ± 11.80 Medical Services (excluding doctors) 76.13 ± 13.41		13.00 ± 8.13	
Years of Service		0.010		0.301
Less than 5 years	72.34 ± 16.15		15.12 ± 11.51	
5 to less than 10 years	72.96 ± 14.04		14.55 ± 8.24	
10 to less than 15 years	79.56 ± 15.11		15.85 ± 11.42	
15 to less than 20 years	70.15 ± 16.12		12.19 ± 9.89	
20 or more years	76.12 ± 13.58		14.95 ± 9.75	

* Pearson’s Correlation Coefficient. PSQ = Perceived Stress Questionnaire; CES-D = Center for Epidemiologic Studies Depression Scale.

**Table 6:** Correlations between spirituality, perceived stress, and depression.

		Spirituality (1)	Perception of Stress (2)	Depression (3)
1	r		−0.479	−0.452
2	r	−0.479		0.674

**Table 7:** Multiple regression analysis with stress as the criterion variable.

	B	Std. Error	t	Sig.
(Constant)	107.104	8.548	12.530	0.000
Gender	3.897	1.842	2.116	0.035
Age	−014	0.110	−124	0.902
Educational level	−1.207	0.939	−1.286	0.200
Service at Hospital	−1.189	1.708	−696	0.487
Sector	1.665	1.137	1.465	0.144
Years of service	0.627	0.642	0.977	0.330
Marital status	−943	0.677	−1.394	0.164
Children	2.010	1.419	1.416	0.158
Spirituality Score	−1.158	0.109	−10.614	0.000
**ANOVA**				
R^2^ = 0.317				
F-ratio = 15.192				
Sig. = 0.000				
N = 304				

Dependent Variables: Perception of Stress Scale (PSQ). Predictors: (Constant), Spirituality Scale, Gender, Marital Status, Educational Level, Sector, Age, Service at Hospital, Children, Years of Service.

## Data Availability

Data supporting this findings are available from the corresponding authors upon reasonable demand.
